# The relationship between glycemic variability and diabetic peripheral neuropathy in type 2 diabetes with well-controlled HbA1c

**DOI:** 10.1186/1758-5996-6-139

**Published:** 2014-12-15

**Authors:** Feng Xu, Li-hua Zhao, Jian-bin Su, Tong Chen, Xue-qin Wang, Jin-feng Chen, Gang Wu, Yan Jin, Xiao-hua Wang

**Affiliations:** Department of Endocrinology, The Second Affiliated Hospital of Nantong University, No. 6 North Hai-er-xiang Road, Nantong, 226001 China; Department of Clinical Laboratory, The Second Affiliated Hospital of Nantong University, No. 6 North Hai-er-xiang Road, Nantong, 226001 China

**Keywords:** Glycemic variability, Continuous glucose monitoring, Diabetic peripheral neuropathy, Type 2 diabetes

## Abstract

**Background:**

Diabetic peripheral neuropathy (DPN) is one of the most common microvascular complications of diabetes. Glycemic variability could be an independent risk factor for diabetes complications in addition to average glucose. Type 2 diabetes with well-controlled glycosylated hemoglobin A1c (HbA1c) may have different terms of glycemic variability and vascular complication consequences. The aim of the study is to investigate the relationship between glycemic variability and DPN in type 2 diabetes with well-controlled HbA1c (HbA1c < 7.0%).

**Methods:**

45 type 2 diabetes with well-controlled HbA1c(HbA1c < 7.0%) and with DPN (DM/DPN group) were recruited in the study, and 45 type 2 diabetes with well-controlled HbA1c and without DPN (DM/–DPN group) were set as controls. The two groups were also matched for age and diabetic duration. Blood pressure, body mass index(BMI), insulin sensitivity index (Matsuda index, ISI), total cholesterol (TC), triglyceride (TG), high density lipoprotein cholesterol (HDLC), and low density lipoprotein cholesterol (LDLC) were tested in the two groups. And all patients were monitored using the continuous glucose monitoring (CGM) system for consecutive 72 hours. The multiple parameters of glycemic variability included the standard deviation of blood glucose (SDBG), mean of daily differences (MODD) and mean amplitude of glycemic excursions (MAGE).

**Results:**

The DM/DPN group had a greater SDBG, MODD and MAGE, when compared to the DM/–DPN group (*p* < 0.05). BMI, TC, and LDLC of DM/DPN group were lower than those of DM/–DPN group (*p* < 0.05). The patients with hypoglycemia were comparable between the two groups (*p* > 0.05). Univariate analysis showed DPN was closely associated with BMI (OR 0.82, CI 0.72–0.94, *p* = 0.005), TC (OR 0.63, CI 0.42–0.93, *p* = 0.02), LDLC (OR 0.4, CI 0.20–0.80, *p* = 0.009), SDBG (OR 2.95, CI 1.55–5.61, *p* = 0.001), MODD (OR 4.38, CI 1.48–12.93, *p* = 0.008), MAGE (OR 2.18, CI 1.47–3.24, *p* < 0.001). Multivariate logistic regression analysis showed that MAGE (OR 2.05, CI 1.36–3.09, *p* = 0.001) and BMI (OR 0.85, CI 0.73–0.99, *p* = 0.033) were significantly correlating with DPN. Glycemic variability, evaluated by MAGE, was the most significantly independent risk factor for DPN.

**Conclusions:**

There was a close relationship between glycemic variability evaluated by MAGE and DPN in type 2 diabetes with well-controlled HbA1c.

## Background

Diabetic peripheral neuropathy (DPN) is one of the most common microvascular complications of diabetes, and is associated with foot ulceration, ampuation and significant reduction in quality of life [1, 2]. DPN affected up to 50% of all diabetic patients with long duration of disease, and the burden of DPN was found to be considerable [3, 4].

The exact pathophysiological mechanisms of DPN in diabetes remain elusive. The potential mechanisms are associated with a number of modifiable and nonmodifiable risk factors, including the degree of hyperglycemia, lipid disorders, high blood pressure, cigarette smoking, alcohol consumption, diabetes duration, height, and so on [5–7]. For all diabetic patients, tight glycemic control is vital important for prevention and treatment of the DPN.

The glycemic disorders in diabetes are not solely limited to fasting and postprandial hyperglycemia, but can be extended to the glycemic variability that includes both upward (postprandial glucose increments) and downward (interprandial glucose decrements) changes [8]. Current diabetic treatments are aimed to control fasting and postprandial glucose levels close to the target in order to prevent the development of diabetes-related complications, with glycosylated hemoglobin A1c (HbA1c) being the gold-standard assessment of long-term overall glycemic control. A reasonable HbA1c level, as defined by the American Diabetes Association(ADA), is below or around 7% [9]. In addition to HbA1c, glycemic variability could be an independent risk factor for diabetes complications [10, 11]. Diabetic patients with target value of HbA1c may have different terms of glycemic variability and vascular complication consequences.

Efforts to quantify glycemic variability have relied on intermittent glucose determinations which acquired from the continuous glucose monitoring (CGM) system. CGM system can detect glycemic variability in more details than the conventional self-monitoring methods of blood glucose [12, 13]. Glycemic variability parameters could be calculated with complex formulas designed specifically for the CGM data.

The present study was designed to determine the relationship between the relationship between glycemic variability assessed by CGM and diabetic peripheral neuropathy in type 2 diabetes with well-controlled HbA1c (HbA1c < 7.0%).

## Materials and methods

### Study subjects

Total 312 type 2 diabetes with diabetic peripheral neuropathy were screened and diagnosed at the inpatient department of the Second Affiliated Hospital of Nantong University from May 2011 to June 2014. The diagnosis of type 2 diabetes was based on the ADA diagnostic criteria 2011 [14]. The diagnosis of DPN was based on the criteria recommended by The Toronto Diabetic Neuropathy Expert Group [15]. Criteria of confirmed DPN included the presence of a symptom or symptoms or a sign or signs of neuropathy and an abnormality of nerve conduction (NC) tests. Symptoms of DPN included decreased sensation, positive neuropathic sensory symptoms (numbness, prickling or stabbing, burning or aching pain, etc.) predominantly in the toes, feet, or legs. Signs of DPN included symmetric decrease of distal sensation or unequivocally decreased or absent ankle reflexes. Signs were revealed through physical examination with tools: touch sensation was tested with a 10-g monofilament on four sites per foot, pain sensation was tested with a pin, reflexes were tested with a tendon hammer, and vibration sensation was tested with a standard 128-Hz tuning fork. The neuropathic deficit of non-diabetic origin (e.g., caused by peripheral vascular disease, arthritis, malignancy, alcohol abuse, vitamin B deficiency, spinal canal stenosis) was excluded through a careful medical history review, a differential test, or both.

And 45 type 2 diabetic patients with well-controlled HbA1c (HbA1c < 7.0%) and with DPN (DM/DPN group) were chosen and recruited for the further study. At the same time, 45 type 2 diabetic patients with well-controlled HbA1c and without DPN (DM/–DPN group) chosen from outpatient were set as controls. The two groups were also matched for age and diabetic duration. All recruited patients had no acute complications such as diabetic ketoacidosis, or other disorders affecting glucose metabolism such as hyperthyroidism. The study was approved by the institutional review board of the Second Affiliated Hospital of Nantong University, with written informed consent being obtained from all participants.

Among DM/–DPN group, 10 patients treated with insulin (6 with basal insulin and 4 with pre-mixed insulin), 23 patients treated with oral hypoglycaemic agents(10 with metformin, 5 with sulfonylureas, and 8 with sulfonylureas and metformin), and 12 patients were on lifestyle intervention. Among DM/DPN group, 17 patients treated with insulin (8 with basal insulin and 9 with pre-mixed insulin), 23 patients treated with oral hypoglycaemic agents (8 with metformin, 8 with sulfonylureas, and 7 with sulfonylureas and metformin), and 5 patients were on lifestyle intervention. Systolic blood pressure (SBP) ≥140 mmHg, or diastolic blood pressure (DBP) ≥90 mmHg, or receiving hypertensive treatment were considered as hypertension. 23 patients in DM/–DPN group were presented with hypertension, and 17 patients in DM/DPN group were presented with hypertension.

### CGM in all subjects

All subjects were monitored by CGM system (Medtronic MiniMed, Northridge, CA 91325, USA) for 72 hours. The CGM system sensor was inserted in all subjects on day 0 and removed on day 3. Data were downloaded and glucose profiles were evaluated based on the data collected on days 1 and 2. The subjects were instructed to input at least four calibration readings per day and the times of key events. During the study, all subjects had standard meals provided by dietary division. The total calorie intake was 30 kcal/kg per day, with 50% carbohydrates, 15% proteins, and 35% fats. The calorie distribution between breakfast, lunch, and dinner was 20%, 40%, and 40%, respectively. Three daily meals were required to consume at time of 6:30 to 7:30, 11:30 to 12:30, and 18:00 to 19:00, respectively. Patients maintained diabetic treatment as usual, and were instructed to avoid strenuous exercise during the CGM.

The parameters of glycemic variability included the standard deviation of blood glucose (SDBG), mean of daily differences (MODD) and mean amplitude of glycemic excursions (MAGE) [16]. MODD that calculated from the absolute difference between paired continuous glucose monitoring values during two successive 24 hour periods was used to assess day-to-day glycemic variability [17]. MAGE, designed to quantify major swings of glycemia and to exclude minor ones, was used for assessing intra-day glycemic variability in this study [18, 19]. Additionally, hypoglycemia was defined as a period with a CGM reading <3.9 mmol/L for at least 15 minutes with an antecedent non-hypoglycemic episode of at least 30 minutes [20].

### Insulin sensitivity index determination

After CGM, blood samples were taken at 0, 30, 60, 90 and 120 min for the measurement of plasma glucose and insulin concentrations (glucose unit: mmol/L, insulin unit: miu/L) during 75-g oral glucose test. Insulin sensitivity was estimated using the insulin sensitivity index (ISI) of Matsuda and DeFronzo: ISI = 10000/square root of (Ins0 × Glu0) × (mean glucose × mean insulin during OGTT) [21].

### Anthropometric indices and laboratory examination

Body mass index (BMI) was calculated (kg/m^2^). SBP and DBP were taken three times using a sphygmomanometer and then were averaged. Capillary glucose concentrations were measured with Lifescan Surestep blood glucose meter. Plasma glucose levels were measured using the glucose oxidase method. HbA1c was measured by high performance liquid chromatography(HPLC) with D-10 hemoglobin Testing Program (Bio-Rad). The serum insulin assay used magnetic beads-based enzymatic spectrofluorometric immunoassay with automatic enzyme immunoassay apparatus (AIA360, TOSOH). Serum glucose concentrations, total cholesterol (TC), triglyceride (TG), high density lipoprotein cholesterol (HDLC), low density lipoprotein cholesterol (LDLC), and serum creatinine(Scr) were measured with Hitachi Model 7600 Series Automatic Analyzer. Glomerular filtration rate(GFR) was estimated by using the reexpressed 4-variable Modification of Diet in Renal Disease (MDRD) Study equation (eGFR = 175 × (standardized Scr) ^–1.154^ × age^–0.203^ × 0.742 [if female]) [22].

### Statistical analyses

Data analyses were performed using the SPSS16.0 statistical software (SPSS Inc., USA). Continuous variables were expressed as means ± standard deviation (SD) or median (interquartile range) in the case of skewed distributions. Categorical variables were described as frequency (percentage). The Student *t*-test was applied to compare differences of continuous variables between the two groups, nonparametric test (Mann–Whitney U test) was applied to compare non-normally distributed variables between the two groups, and Chi-squared test was applied to compare categorical variables between the two groups. Two logistic regression analysis were performed to assess the impact of different risk factors on DPN: the univariate analysis were performed to estimate the contribution of clinical risk factors to DPN using the odds ratio (OR) and 95% confidence interval (CI), and the multivariate logistic regression analysis were conducted to identify independent risk factors for DPN. The risk factors were selected by a forward selection procedure based on increment of R^2^ in the multivariate logistic regression analysis. *p* < 0.05 was considered to be statistically significant.

## Results

### Baseline characteristics in the subjects

As shown in Table 1, age, sex distribution, diabetic duration, SBP, DBP, TG, HDLC, eGFR, HbA1c, and Matsuda ISI were comparable between DM/–DPN and DM/DPN groups (*p* > 0.05). 22.2% (n = 10) treated with insulin, 51.1%(n = 23) treated with oral hypoglycaemic agents, and 26.7%(n = 12) was on lifestyle intervention in DM/–DPN group; 37.8% (n = 17) treated with insulin, 51.1% (n = 23) treated with oral hypoglycaemic agents, and 11.1% (n = 12) was on lifestyle intervention in DM/DPN group. The two groups were comparable with regard to insulin treatment, oral hypoglycaemic agents, lifestyle intervention and stain medication (*p* > 0.05). BMI, TC, and LDLC of DM/DPN group were lower than those of DM/–DPN group (*p* < 0.05). The current drinking and smoking were comparable between the two groups (*p* > 0.05). The prevalence of hypertension was 51.1% in DM/–DPN group, compared with 37.8% in DM/DPN group (*p* > 0.05).

**Table 1:** **Comparisons of clinical variables between DM/–DPN and DM/DPN groups**

	DM/–DPN group	DM/DPN group	*t*/ χ ^2^	*p*
n	45	45	–	–
Age (year)	58.7 ± 6.6	59.8 ± 8.3	0.675	0.501
Female, n (%)	24 (53.3)	20 (44.4)	0.711	0.399
Diabetic duration (year)	5.0 (2.0–8.0)	6.0 (2.0–9.0)	**–**	0.413
Insulin treatment, n (%)	10 (22.2)	17 (37.8)	2.593	0.107
Lifestyle intervention, n (%)	12 (26.7)	5 (11.1)	3.554	0.059
Hypertension, n (%)	23 (51.1)	17 (37.8)	1.620	0.203
Stain medication, n (%)	8 (17.8)	13 (28.9)	1.553	0.213
Current drinking, n (%)	20 (44.4)	22 (48.9)	0.179	0.673
Current smoking, n (%)	17 (37.8)	20 (44.4)	0.413	0.520
BMI (kg/m^2^)	26.2 ± 3.9	23.9 ± 3.3	3.123	0.002
Height (m)	1.68 ± 0.07	1.66 ± 0.08	0.921	0.360
SBP (mmHg)	139 ± 18	134 ± 18	1.317	0.191
DBP (mmHg)	83 ± 12	80 ± 10	1.296	0.198
TG (mmol/L)	1.6 (1.0–2.9)	1.1 (0.8–1.9)	**–**	0.057
TC (mmol/L)	5.0 ± 1.3	4.4 ± 1.0	2.482	0.015
HDLC (mmol/L)	1.2 ± 0.3	1.1 ± 0.3	1.815	0.073
LDLC (mmol/L)	2.7 ± 0.8	2.3 ± 0.6	2.839	0.006
eGFR (ml/min/1.73 m^2^)	110 ± 26	105 ± 22	0.706	0.482
Matsuda ISI	94(69–145)	110 (58–153)	**–**	0.707
HbA1c (%)	6.4 ± 0.4	6.5 ± 0.4	1.576	0.119
Hypoglycemia, n (%)	3 (6.7)	6 (13.3)	1.111	0.292
SDBG (mmol/L)	2.1 ± 0.6	2.8 ± 0.9	3.800	<0.001
MODD (mmol/L)	1.9 ± 0.3	2.2 ± 0.6	2.873	0.005
MAGE (mmol/L)	4.5 ± 0.9	5.8 ± 1.6	2.839	<0.001

Normally distributed values in the table are given as the mean ± SD, non-normally distributed values are given as the median (25% and 75% interquartiles), and categorical variables are given as frequency (percentage).

DM/–DPN group: well-controlled type 2 diabetes without DPN; DM/DPN group: well-controlled type 2 diabetes with DPN.

BMI: body mass index; SBP/DBP: systolic/diastolic blood pressure; TC: total cholesterol; TG: triglyceride; HDLC: high density lipoprotein cholesterol; LDLC: low density lipoprotein cholesterol; HbA1c: glycosylated hemoglobin A1c; ISI: insulin sensitivity index; eGFR: estimated glomerular filtration rate; SDBG: standard deviation of blood glucose; MODD: mean of daily differences; MAGE: mean amplitude of glycemic excursions.

### Glycemic variability in the subjects

The glycemic variability parameters from CGM data were shown in Table 1. The DM/DPN group had a greater SDBG (2.8 ± 0.9 vs. 2.1 ± 0.6 mmol/L, *p* < 0.001), MODD(2.2 ± 0.6 vs. 1.9 ± 0.3 mmol/L, *p* = 0.005) and MAGE (5.8 ± 1.6 vs. 4.5 ± 0.9 mmol/L, *p* < 0.001), when compared to the DM/–DPN group. 6.7% (n = 3) in DM/–DPN group had a total of 4 hypoglycemic events, meanwhile 13.3% (n = 6) in DM/DPN group had a total of 10 hypoglycemic events (6.7% vs. 13.3%, *p* > 0.05).

### Relationships between multiple risk factors and DPN by univariate and multivariate analysis

In this study, the univariate analysis showed DPN was closely associated with BMI(OR 0.82, CI 0.72–0.94, *p* = 0.005), TC (OR 0.63, CI 0.42–0.93, *p* = 0.02), LDLC (OR 0.4, CI 0.20–0.80, *p* = 0.009), SDBG (OR 2.95, CI 1.55–5.61, *p* = 0.001), MODD (OR 4.38, CI 1.48–12.93, *p* = 0.008), MAGE(OR 2.18, CI 1.47–3.24, *p* < 0.001). And DPN failed to associate with age, diabetic duration, hypertension, insulin treatment, lifestyle intervention, stain medication, current drinking, current smoking, TG, HDLC, HbA1c, eGFR, Matsuda ISI (*p* > 0.05) (Table 2). Multivariate logistic regression analysis showed that MAGE(OR 2.05, CI 1.36–3.09, *p* = 0.001) and BMI(OR 0.85, CI 0.73–0.99, *p* = 0.033) were significantly correlating with DPN(Nagelkerke R^2^ = 0.317) (Table 2). Glycemic variability, evaluated by MAGE, was the most significantly independent risk factor for DPN.

**Table 2:** **Relationships between multiple risk factors and DPN, by univariate and multivariate analysis**

Variable	Univariate analysis (OR; 95% CI)	*p*	Multivariate analysis (OR; 95% CI)	*p*
Age (year)	1.02 (0.96–1.08)	0.497	–	
Female, n (%)	1.43 (0.62–3.28)	0.400	–	
Diabetic duration (year)	1.05 (0.97–1.15)	0.224	–	
Insulin treatment, n (%)	2.13 (0.84–5.36)	0.111	–	
Lifestyle intervention, n (%)	0.34 (0.11–1.08)	0.067	–	
Hypertension, n (%)	0.58 (0.25–1.35)	0.205	–	
Stain medication, n (%)	1.88 (0.69–5.11)	0.216	–	
Current drinking, n (%)	1.20 (0.52–2.74)	0.673	–	
Current smoking, n (%)	1.32 (0.57–3.06)	0.521	–	
BMI (kg/m^2^)	0.82 (0.72–0.94)	0.005	0.85(0.73–0.99)	0.033
Height (m)	0.97 (0.92–1.03)	0.356	–	
TG (mmol/L)	0.80 (0.62–1.04)	0.097	–	
TC (mmol/L)	0.63 (0.42–0.93)	0.020	–	
HDLC (mmol/L)	0.26 (0.06–1.16)	0.078	–	
LDLC (mmol/L)	0.40 (0.20–0.80)	0.009	–	
eGFR (ml/min/1.73 m^2^)	1.00 (0.98–1.01)	0.478	–	
Matsuda ISI	1.04 (0.59–1.85)	0.891	–	
HbA1c (%)	2.45 (0.79–7.64)	0.122	–	
Hypoglycemia, n (%)	2.15 (0.50–9.21)	0.301	–	
SDBG (mmol/L)	2.95 (1.55–5.61)	0.001	–	
MODD (mmol/L)	4.38 (1.48–12.93)	0.008	–	
MAGE (mmol/L)	2.18 (1.47–3.24)	<0.001	2.05 (1.36–3.09)	0.001

Results are given as odds ratios and 95% confidence intervals (OR; 95% CI).

Nagelkerke R^2^ = 0.317 in multivariate analysis.

## Discussion

HbA1c is not correlated with glycemic variability in well-controlled type 2 diabetes [23], and diabetic patients with target value of HbA1c may have different terms of glycemic variability. Hay et al. [24] reported that excessive postprandial glycemic excursions were common in well-controlled patients with type 2 diabetes treated with a sulfonylurea with or without metformin. Our previous study also showed that a segment of type 2 diabetes treated with insulin and with well-controlled HbA1c demonstrated elevated glycemic excursions [25]. In the present study DPN patients with well-controlled HbA1c showed a higher glycemic variability, compared to the matched type 2 diabetes with well-controlled HbA1c and without DPN.

We also evaluated and compared the effect of the control of glycemic variability on the development of DPN in well-controlled type 2 diabetes in the study. The results showed that there were close relationships between glycemic variability parameters and DPN in type 2 diabetes. Several previous studies showed the fasting plasma glucose(FPG) and HbA1c variability and the risk of microvascular complications in diabetes [26–29], but seldom studies showed glycemic variability accessed by CGM and microvascular complications [30, 31]. Takao et al. [26, 27] revealed that in type 2 diabetes FPG variability can predict diabetic retinopathy development and progression independently of the mean FPG or HbA1c. Lin et al. [28] showed annual FPG and HbA1c variability had a strong association with diabetic nephropathy in type 2 diabetes. Kilpatrick et al. [29] in their study showed that variability in HbA1c added to the mean value in predicting of retinopathy and nephropathy in type 1 diabetes. Sartore et al. [30] showed that glycemic variability, expressed by CGM-derived indicators of short-lived glycemic fluctuations, was an important part of glycemic control in relation to the prevalence of diabetic retinopathy in both type 1 and type 2 diabetes. In a pilot study, Oyibo et al. [31] showed patients with painful neuropathy had greater glycemic excursions and possibly poorer diabetes control, compared with patients with painless neuropathy. Our study strengthens the evidence base that glycemic variability, accessed by CGM, is associated with microvascular complications among type 2 diabetes. And to our knowledge, this is the first study to document that glycemic variability accessed by CGM may play an important role in the development of DPN in type 2 diabetes, in spite of these patients with well-controlled HbA1c.

In the present study multivariate regression analysis showed that glycemic variability, evaluated by MAGE, was the most significantly correlating with DPN (OR 2.05, CI 1.36–3.09, *p* = 0.001). MAGE was considered as a well-validated index of glycemic variability in the paper of Monnier et al. [19], and activation of the oxidative stress by MAGE and overproduction of mitochondrial superoxide may play an axile role in the pathogenesis of diabetic complications [32, 33]. Several studies had demonstrated the association between glycemic variability evaluated by MAGE and macrovascular complications. Torimoto et al. [34] documented that MAGE played a significant role in vascular endothelial dysfunction and in progression of atherosclerosis in type 2 diabetes. Su et al. [35] documented MAGE was associated with the presence and severity of coronary artery disease in type 2 diabetes. MAGE may be an important predictor of mortality and major adverse cardiac event (MACE) in elderly patients after acute myocardial infarction (AMI) [36], and elevated admission MAGE appeared more important than admission glucose and prior long-term abnormal glycometabolic status in predicting 1-year MACE in patients with AMI [37]. In our study, the close association between MAGE and DPN(microvascular complication) was documented. Increased MAGE could result in an increased risk for both microvascular and macrovascular complications.

Several variables such as obesity, increased height, presence of hypertension, antidiabetic treatment type, current smoking and drinking, lipid disorders (such as elevated TC, TG and LDLC), identified as predictors of DPN in other populations [6, 7], did not emerge as independent predictors in the present study. According to our results patients with DPN had a significantly lower BMI, TC and LDLC, and lower BMI was the independent risk for DPN in the multivariate regression analysis. Lean (lower BMI), lower TC or LDLC may imply imbalance of nutrition in patients with DPN, which may not benefit to the rehabilitation of DPN. And moderate BMI and balanced nutrition may promote the rehabilitation of DPN. Lower BMI may be a new potential independent risk factor for DPN. There was a controversy in the relationship between insulin therapy and presence of DPN. Katulanda et al. [38] showed there was a significant association between the use of insulin and presence of DPN. Pop-Busui et al. [39] showed a glycemic control therapy with insulin-sensitizing significantly reduced the incidence of DPN compared with insulin-providing therapy among patients with type 2 diabetes followed for up to 4 years during the study. Our study showed that presence of DPN did not associated with insulin therapy. The reason may be that the study populations were different in sample, ethnicity, diabetic duration, glycemic status, and so on.

It should be pointed out that our study had some limitations. First, the most obvious limitation of the study was the cross-sectional statistical analysis, which only analyzed the relationship between magnitude of glycemic variability and DPN, and could not analyze long-term of glycemic variability and DPN. Second, it needs a follow-up study to investigate the admission MAGE in the role of the improvement of DPN. If the result is positive, it could further strength the close relationship between glycemic variability and DPN. Third, we could not assess the relationship between oxidative stress or inflammation and glycemic variability. Fourth, although we provided standard meals for patients and maintained the patients’ diabetic treatment as usual during the CGM system monitoring period, some factors, such as physical activity and emotional stress, etc., which may affect levels of glycemic variability, could not all be prevented.

## Conclusion

In summary, DPN patients with well-controlled HbA1c showed a higher glycemic variability, compared with well-controlled type 2 diabetes without DPN. And glycemic variability, evaluated by MAGE, was the significantly independent risk factor for DPN in type 2 diabetes with well-controlled HbA1c (HbA1c < 7.0%).

## Abbreviations

DPN: Diabetic peripheral neuropathy
BMI: Body mass index
SBP/DBP: Systolic/diastolic blood pressure
TC: Total cholesterol
TG: Triglyceride
HDLC: High density lipoprotein cholesterol
LDLC: Low density lipoprotein cholesterol
HbA1c: Glycosylated hemoglobin A1c
ISI: Insulin sensitivity index
eGFR: Estimated glomerular filtration rate
SDBG: Standard deviation of blood glucose
MODD: Mean of daily differences
MAGE: Mean amplitude of glycemic excursions.
